# Techno-economic and sustainability assessment of the power to MeOH processes: The present and future perspective

**DOI:** 10.1016/j.heliyon.2024.e39860

**Published:** 2024-10-26

**Authors:** Ali Pakdel, Reza Eslamloueyan

**Affiliations:** Chemical Engineering Department, School of Chemical and Petroleum Engineering, Shiraz University, Iran

**Keywords:** Power-to-X, Sustainability, CO_2_ hydrogenation, Economic assessment, Methanol production

## Abstract

This study presents a comprehensive analysis of a Power-to-Methanol (PtM) process, which converts CO_2_ and H_2_ derived from electrolysis of water into methanol (MeOH) using renewable energy sources. The process is analyzed for its technical, economic, and environmental aspects, focusing on production costs and the minimum selling price of MeOH. The simulation results show an energy efficiency of 56 % in the optimal state, with a CO_2_ consumption of 1.4 kg-CO_2_/kg-MeOH. Economic evaluations for 2023 indicate production costs of US$ 631 and 643 for PtM processes equipped with membrane and amine absorption technologies, respectively. The study also predicts the cost of production and minimum selling price of MeOH from 2023 to 2030 based on the trends of electrolyzer capital investment and renewable resource prices. The results provide valuable insights for the development and evaluation of sustainable MeOH production processes.

## 1 Introduction

**Fig. 1:**
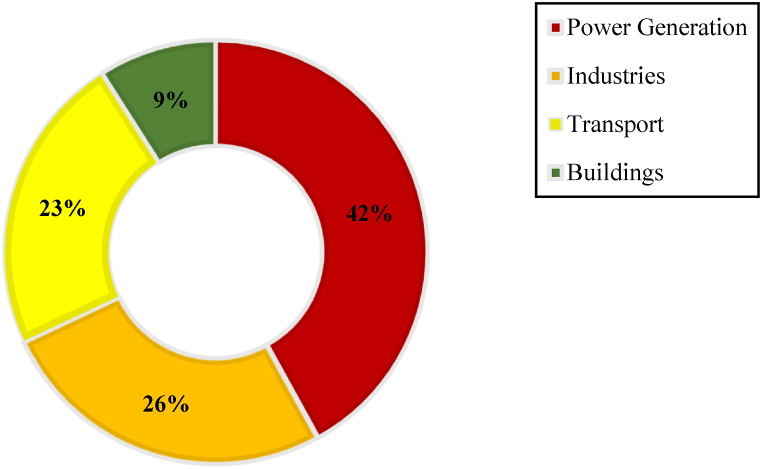
Contribution of different sources of carbon dioxide emissions to the atmosphere [4].

In 2015, the United Nations conference was held in Paris (COP21), and the participating countries were bound to reduce greenhouse gas emissions (GHG) in the energy sector to zero by 2050, or control their greenhouse gas emissions by 2030 [1]. Greenhouse gases are a significant concern for the planet's health. They trap heat in the atmosphere, leading to global warming and climate change [2]. The most common greenhouse gases are carbon dioxide (CO_2_), methane (CH_4_), Sulfur dioxide (SO_2_), and nitrous oxide (N_2_O) [3]. These gases are emitted into the atmosphere by burning fossil fuels, deforestation, and industrial processes. Today, we can no longer deny the reality of climate change and global warming due to the emission of greenhouse gases, particularly CO_2_. We must find ways to remove this pollutant from the atmosphere. According to the International Energy Agency’s report, the total carbon dioxide emissions in various sectors in 2022 were approximately 36.8 gigatons. Fig. 1 shows the production share of each sector. The power generation sector is responsible for emitting the highest amount of polluting emissions, producing 14.7 gigatons of CO_2_ annually. Various industries, such as chemical and metal industries, are in the next rank. The transportation sector has a 23 % share in pollution emissions, and the heating and cooling of buildings are in the last rank [4].

Meanwhile, in 2023, carbon dioxide emissions had risen to 43.7 gigatons, which can increase the average temperature of the Earth [5]. It is expected that with the increase in energy demand and population growth, this amount will increase in the coming years. If the amount of greenhouse gas pollution is not controlled and increases according to the current trends, it will have irreparable consequences.

Since our research is focused on PtM process, in the following we have presented a complete review of the research works done in this field. MeOH is a basic petrochemical product with numerous applications across various industries, making it one of the most extensively used substances. The production capacity of MeOH in the world in 2023 is reported to be about 100 million tons [6]. The production cost of each ton of MeOH produced from coal and fossil resources is reported to be around US$ 100–250 per ton [7]. Conventional MeOH plants release approximately 1.5 tons of carbon dioxide per ton of MeOH production [8]. Each year, MeOH production plants release about 150 million ton of CO_2_ into the atmosphere, which is a significant amount. According to the mentioned facts, replacing the current MeOH plants with new MeOH production technologies having very low carbon dioxide emissions has attracted the interest of many researchers. Research in the field of MeOH production with the PtX process was conducted in 2023 by Sollai et al. They developed a MeOH production process with a capacity of 500 kg/h using renewable resources and carbon dioxide absorbed from a power plant. For the mentioned production capacity, a polymer membrane electrolyzer with a power of 6 MW was used. Based on their techno-economic assessments, the cost of MeOH production was reported as EU€ 960 per ton of MeOH [9]. In 2024, Li et al. analyzed the thermodynamic, economic, and CO_2_ emission aspects of production process of MeOH from renewable resources. The process includes alkaline electrolyzer system for producing H_2_, CO_2_ capturing unit and MeOH synthesis unit, the energy and exergy efficiency of the simulated process were reported as 36 % and 30 %, respectively, while the cost of MeOH production in this process reached US$ 840 per ton [10]. In 2024 Fogel et al. conducted a techno-economic assessment to ascertain the specific production costs of renewable MeOH. The specific methanol production costs for small-scale applications (1.12 MW solid oxide electrolyzer) were determined to be € 2419 per ton of MeOH, representing a more than fourfold increase over the prevailing market price of conventionally produced MeOH. The study revealed that increases in system scale led to decreased MeOH production costs due to economy-of-scale effects. Furthermore, the sensitivity of the process economics was evaluated to key operational and capital characteristics [11]. Martsinchyk et al. conducted a feasibility analysis of a power-to-gas system, examining various operating points and capacities. The study utilized a system model that included a solid oxide electrolyzer, a CO_2_ separation unit, and a methanation reactor as the main components. The study suggests that in a solid oxide electrolyzer-based power-to-gas system, the cost of synthetic natural gas (SNG) production is expected to decrease by 15–21 % by 2030 and 29–37 % by 2050 [12].

While various articles have been published in recent years on the price of MeOH produced by the PtX process, the impacts of advancements in renewable energy technologies and water electrolysis methods on the price of this substance have not been thoroughly and quantitatively examined. Therefore, in this study, we comprehensively discuss this topic. Four major novelties can be mentioned for our research work: (1) designing and simulating a PtM process with a capacity of an average conventional MeOH plant, (2) optimizing the MeOH synthesis reactor based on the feed conditions, (3), conducting heat integration for the proposed process to minimize the energy consumption, and (4) evaluating the developed PtM process from technical, economic, and environmental perspective. We have also discussed the possibility of replacing conventional MeOH plants with PtM processes in the future.

## 2 Methodology

This section describes the PtM process and the basis of the process simulation and modeling in Aspen HYSYS software. Also, the optimization algorithm and economic and environmental criteria are discussed.

### 2.1 Process description

The process developed in this research for the annual production of 82000 tons of MeOH (this capacity is in the order of a conventional MeOH production plant) consists of two parts: (1) the hydrogen production unit, and (2) the MeOH production and purification units, which are designed and simulated in Aspen HYSYS software under steady-state conditions. The hydrogen production unit includes an alkaline electrolyzer, a centrifugal pump, a heat exchanger, and two-phase separators. The MeOH production and purification units consist of five equipment items.

• Centrifugal compressors and pumps
• Shell and tube heat exchangers
• A MeOH synthesis reactor
• Two-phase separators
• A sieve-tray distillation tower

The CO_2_ capturing unit has not been designed and simulated in this work, and it is assumed that this component is captured in other plant and transferred as feedstock to the PtM production unit.

#### 2.1.1 Hydrogen production unit

**Fig. 2:**
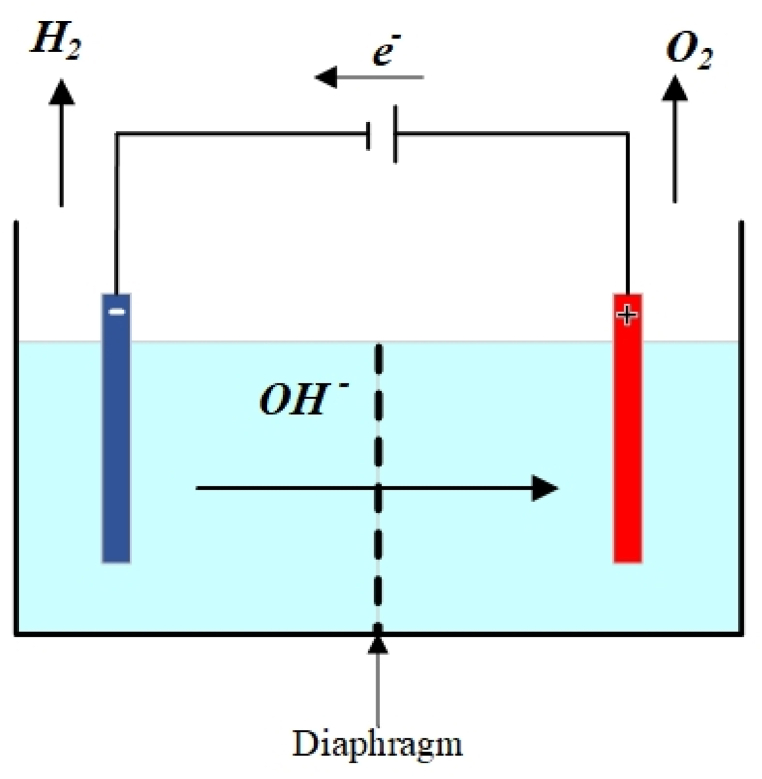
Alkaline electrolyzer operation diagram [1].

Hydrogen is a crucial element in the manufacturing of fuels and chemicals [13]. The most common procedure for producing hydrogen is Steam Methane Reforming (SMR) [14], but unfortunately, this process emits approximately 7 tons of carbon dioxide for each ton of hydrogen produced [15]. Electrolysis of water using renewable resources is another hydrogen production process that releases significantly less CO_2_ than SMR [16]. There are various types of electrolyzers, such as alkaline, polymer membrane, and solid oxide electrolysis cells (SOEC) [17]. However, SOEC has yet to be used on an industrial scale. Fig. 2 illustrates how the alkaline electrolyzer functions. By the flow of electrons from the anode to the cathode and the consumption of H^+^ ions, hydrogen is produced on the cathode side. OH^−^ ions are oxidized by passing through the diaphragm in the water and KOH solution on the anode side, which produces water and oxygen [18].

An alkaline electrolyzer supplies hydrogen to the PtX process, which produces hydrogen using renewable electricity according to the following reactions in the cathode and anode [1].

Cathode side:

(1) $${2H}_{2}O+2e^{-}\;↔H_{2}+2{OH}^{-}$$

Anode side:

(2) $$2{OH}^{-}\;↔H_{2}O+0.5O_{2}+2e^{-}$$

Overall reaction:

(3) $$H_{2}O\;↔H_{2}+0.5O_{2}$$

Today, alkaline electrolyzers are facing challenges that can be pointed to the stability and very high cost of production. The high rate of destruction of catalyst and electrodes in the electrolytic environment has caused electrolyzers to be used mostly in laboratories and small scale and rarely used in industry [19].

#### 2.1.2 CO_2_ capturing

Nowadays, there are various commercial technologies that capture CO_2_ from a unit's flue gas or directly from the atmosphere. Among these technologies, we can mention two methods: (1) chemical absorption with amine solutions [20], and (2) membrane separation [21]. There are certain drawbacks associated with each of these approaches. The first method requires a large amount of energy to absorb carbon dioxide since the process involves a distillation tower. Therefore, from an economic point of view, the absorption of CO_2_ by amine solutions has a high operating cost. The production of a membrane suitable for carbon dioxide separation makes the second method expensive due to its complexity. A suitable solution that can absorb CO_2_ with a low operational and capital costs is still under investigation. This study did not model the CO_2_ absorption unit, which is assumed to be the feedstock for the PtX process. Only the cost of CO_2_ capturing per ton, extracted from available literature, was included in the economic analyses. To achieve the specified MeOH production capacity, a stream capable of 14.1 metric tons of carbon dioxide per hour is imperative. In Table 1, the cost of carbon dioxide capture for different technologies is presented.

**Table 1:** CO_2_ capturing cost for different technologies.

Capturing Technology	*Cost, US$/*${ton}_{{CO}_{2}}$	Ref.
*Amin solution*	*30–60*	[20]
*Membrane separation*	*20–30*	[21]

#### 2.1.3 MeOH synthesis and purification unit

Fig. 5 demonstrates the proposed PtM process flow diagram. The hydrogen feed mixes with the captured carbon dioxide stream at a molar ratio of 3:1 at a pressure of 30 bar and a temperature of 80 °C. The mixture after passing through a compressor, which has 1700 kW power and 85 % isentropic efficiency, reaches the pressure of 8472 kPa, mixes with the recycled flow from the two stages of flash separation. The heat exchangers preheat the resulting stream up to the MeOH reactor's optimal design temperature. The heated gas stream then enters a fixed-bed catalytic reactor in which MeOH synthesis reactions take place. The reactor outlet stream goes to the separation unit and the MeOH and water products are separated and the unreacted gases recycled to the reactor inlet.

##### 2.1.3.1 Reaction network and kinetic model

The commercial catalyst bed, commonly used in MeOH synthesis, is composed of CuO/ZnO/Al_2_O_3_. The kinetic model proposed by Van den Bussche and Froment (VBF) was employed in this study for modeling of the reactor [22]. The VBF kinetics comprises two reversible reactions, as given below:

(4) $${CO}_{2}+3H_{2}\;↔{CH}_{3}OH+H_{2}O\Delta H=-49\;kJ/mol$$

(5) $${CO}_{2}+H_{2}\;↔CO+H_{2}O\Delta H=+41\;kJ/mol$$

The first reaction is the main reaction of synthesis gas conversion to MeOH. Here, MeOH is produced from the direct hydrogenation of carbon dioxide, which is an exothermic and reversible reaction. Theoretically, the increase in temperature causes an increase in the rate of exothermic reaction and, as a result, more product formation. In contrast, this increase in temperature causes a decrease in the equilibrium conversion of the reversible reactions [23]. To overcome the inappropriate effect of temperature increase, the reaction should be carried out at high pressure so that both the conversion and the reaction rate of MeOH synthesis are high so that the reaction takes place in a feasible residence time. The side reaction that takes place on the surface of this catalyst is known as reverse water-gas shift, which is endothermic, and increasing the temperature does not have a bad effect on the equilibrium conversion. Increasing the temperature leads to a higher rate of the side reaction, which consumes reactants and results in less MeOH production. The proposed reaction rates are as follows [22]:

(6) $$r_{CO_{2}}=\frac{k_{CO_{2}}P_{CO_{2}}P_{H_{2}}-k_{CO_{2}}P_{MeOH}P_{H_{2}O}P_{H_{2}}^{-2}K_{eq,CO_{2}}^{-1}}{{\left[1+K_{H_{2}}^{0.5}P_{H_{2}}^{0.5}+K_{H_{2}O}P_{H_{2}O}+K_{H_{2}O}K_{H_{2}}^{-1}P_{H_{2}O}P_{H_{2}}\right]}^{3}}$$

(7) $$r_{rwgs}=\frac{k_{rwgs}P_{CO_{2}}-k_{rwgs}P_{CO}P_{H_{2}O}P_{H_{2}}^{-1}K_{eq,rwgs}^{-1}}{\left[1+K_{H_{2}}^{0.5}P_{H_{2}}^{0.5}+K_{H_{2}O}P_{H_{2}O}+K_{H_{2}O}K_{H_{2}}^{-1}P_{H_{2}O}P_{H_{2}}\right]}$$

According to the Arrhenius equation, the reaction rate constant is defined as follows:

(8) $$k_{i}=k_{0,i}\;\exp\left(\frac{-E_{i}}{RT}\right)$$

which in Eq. (8), $k_{0,i}$ is the frequency factor of *i*th reaction, *E*_*i*_ is *i*th reaction activation energy, *R* and *T* are universal gases constant and temperature, respectively. Adsorption constants for each component are calculated from the following equation.

(9) $$K_{j}=A_{0,j}\;\exp\left(\frac{\Delta H_{j}}{RT}\right)$$

In Eq. (9), $A_{0,j}$ is adsorption constant of *j*th component in reference temperature, and $\Delta H_{j}$ is the adsorption enthalpy of *j*th component. Table 2 shows the parameters of the VBF model.

**Table 2:** VBF kinetic model parameters [24].

Parameter	Value
$k_{0,CO_{2}}$, *1/s*	*1.07*
$k_{0,rwgs}$, *1/s*	*1.22×10*^*10*^
$E_{CO_{2}}$, *kJ/mol*	*36.70*
$E_{rwgs}$, *kJ/mol*	*94.77*
$A_{0,H_{2}}$, 1/bar	*6.62×10*^*−*^*^11^*
$A_{0,H_{2}O}$,1/bar	*0.25*
$\Delta H_{H_{2}}$, *kJ/mol*	*34.40*
$\Delta H_{H_{2}O}$, *kJ/mol*	*124.12*

The equilibrium constants of VBF model reactions are calculated from the following expressions.

(10) $$\log_{10}K_{eq,CO_{2}}=\frac{3066}{T}-10.59$$

(11) $$\log_{10}K_{eq,rwgs}=\frac{-2073}{T}+2.03$$

The data obtained from an industrial unit's MeOH production reactor [25] was used to validate the VBF model. The reactor feed conditions of the industrial reactor are described in Table 3.

**Table 3:** Feed conditions of the industrial MeOH reactor [25].

T = 230 °C; P = 7800 kPa	
*Component*	*Molar flow [kmol/h]*
*CO*	*335.81*
*CO*_*2*_	*643.46*
*H*_*2*_	*3825.44*
*H*_*2*_*O*	*5.07*
*CH*_*3*_*OH*	*20.72*
*Inert (N*_*2*_*, CH*_*4*_*)*	*1978.06*

##### 2.1.3.2 MeOH synthesis reactor

Maintaining the optimal temperature profile is crucial in the design of the MeOH synthesis reactors. In this study, LURGI company's reactor technology, known as Boiling Water Reactor (BWR), was used [24].

In this reactor, which is like a shell-and-tube heat exchanger, catalyst pellets are loaded in the reactor tubes. The synthesis gas passing through these tubes reacts in the pores of the catalyst. Saturated water enters the reactor shell, which turns into saturated steam by removing the heat of reaction. This heat removal controls the temperature of the reaction, and along the reactor tubes, as the temperature of the synthesis gas decreases, the reaction moves away from its equilibrium, and more MeOH is produced. To simulate the MeOH synthesis reactor, the multi-tubular plug flow reactor was used. The Ergun equation was used to calculate the pressure drop in the reactor's tubes. The reactor shell-side operates in isothermal mode. The fixed design parameters of the MeOH synthesis reactor are given in Table 4.

**Table 4:** MeOH reactor design parameters [25].

Parameter	Value/Comment
*Type*	*Quasi-Isothermal*
*tube length, m*	*6.00*
*tube diameter, mm*	*38.10*
*void fraction, -*	*0.40*

The length and thickness of the MeOH production reactor tubes are similar to the industrial unit reactor. In contrast, the optimal values of the temperature and pressure of the reactor inlet are determined using optimization.

##### 2.1.3.3 MeOH separation and purification

The reactor’s outlet stream consisting of MeOH, water and unreacted synthesis gas is cooled to 55 °C and enters the first stage flash drum with a diameter of 1.4 m and a length of 4.8 m, where the synthesis gas exits from the top, and the liquid mixture of water and MeOH exits from the bottom of the separator. The pressure of the liquid stream is reduced to a pressure of 150 kPa and enters the second stage flash drum, which has a diameter and length of 1 and 5 m, respectively. Unreacted gases separated from the first and second flash drums are compressed and recycled to the reactor inlet. The 2nd stage separator bottom stream is fed to the distillation tower at a pressure of 150 kPa and a temperature of 55 °C. Pure MeOH exits from the top of the tower, while water product exits from the bottom of the tower. The specifications of the MeOH purification distillation tower are listed in Table 5.

**Table 5:** Distillation column design parameters.

Parameter	Value/Comment
*trays Type*	*Sieve*
*trays count*	*40*
*feed tray*	*18*
*Reflux ratio*	*1.00*
*condenser type*	*Total*
*Reboiler type*	*Kettle*
*Tower diameter, m*	*2.40*
*Tower height, m*	*29.30*

The Fenske-Underwood-Gilliland shortcut method was utilized to find the number of equilibrium stages and the optimal feed tray for the MeOH purification distillation column [26].

### 2.2 Process simulation and design

The proposed PtM plant was modeled and simulated at steady state conditions by the Aspen HYSYS software. The Peng-Robinson equation of state was chosen to estimated gas and liquid properties and VLE calculations in the MeOH synthesis and separation unit, while in the electrolyzer section, the ENRTL-RK fluid package was employed in thermodynamic calculations. To maximize MeOH production, the reactor design conditions were optimized by the Particle Swarm Optimization (PSO) algorithm. The optimizing function was programed in MATLAB environment and it was linked to the process simulation in Aspen HYSYS software. The final optimum design was assessed based on economic, environmental, and technical criteria.

### 2.3 Optimization by PSO algorithm

The PSO algorithm was developed by Kennedy and Eberhart by taking the example of a group swimming of fish and a group flight of birds. In this algorithm, first, a random population of individuals is formed, which is called a group of particles. Each particle in the group has its own set of characteristics that require optimization of their respective parameters. In this method, every particle represents a point in the problem-solving space. The inherent memory of particles enables them to retain the optimal point they reach during the search process within a given space. Each particle can move in two different directions: 1- towards the best position, they have personally reached, and 2- The best position that the group of particles has reached. The knowledge and experience of each particle and other particles change each particle's position in the search space in this algorithm [27].

The reactor design parameters, called decision variables, can be categorized into two groups: (1) operating conditions and (2) reactor structure. The first decision variable group consists of the feed temperature, feed pressure, the reactor shell-side temperature, and the molar ratio of H_2_ and CO_2_ in the reactor feed. The second decision variable group is related to the reactor geometry and structure. Some of these variables are determined based on the engineering heuristics. For instance, the tube diameter and length of the MeOH reactor are selected according to conventional industrial reactors, as shown in Table 4. However, the number of reactor tubes (NoT) as a decision variable is determined through the optimization procedure. The ranges of decision variables considered for the reactor optimization are as follows:

(12) 100<NoT<3000

(13) $$200<T_{in}<300\;\left[{}^{\circ}C\right]$$

(14) $$4500<P_{in}<9000\;\left[kPa\right]$$

(15) $$215<T_{BW}<265\;\left[{}^{\circ}C\right]$$

(16) $$0.5<H_{2}/CO_{2}<4$$

in this study, the molar flow of MeOH at the reactor's outlet is considered as the objective function, and efforts were made to maximize its value.

(17) $$OF=-F_{Out,MeOH}$$

The population generated by the algorithm are sent as decision variables to the Aspen Hysys, and here the process is simulated with these variables and the value of the selected objective function is determined. This value is sent to MATLAB and it is evaluated that if this value is the optimal state of the objective function, the algorithm will report the answer as the solution to the problem, otherwise, the process of creating a random population will be done again by the algorithm and the variables will be sent to the Aspen Hysys to determine the optimal value of the objective function.

### 2.4 Heat exchanger network

The heating and cooling potential of the process streams can be used to reduce the consumption of hot and cold utilities [28]. For example, hot process streams can be used instead of steam to boil the liquid entering the tower reboiler. This allows us to avoid producing carbon dioxide due to utility production. The process was simulated in Aspen HYSYS software and then exported to Aspen Energy Analyzer software for thermal analysis and heat integration. Here, the cooling water temperature is 30 °C, and the minimum approach temperature is 15 °C. The heat exchangers are shell-and-tube types, and they are all rigorously designed and simulated using Aspen Exchanger Design and Rating (EDR) software.

### 2.5 Process evaluation indicators

This sub-section introduces and describes various indices used for evaluating the sustainability of the designed PtM process.

#### 2.5.1 The process energy efficiency

In simple terms, a process's energy efficiency equals the ratio of heat and work produced to heat and work consumed in the process. When it comes to PtX processes, a significant portion of the energy consumed is attributed to the electrolyzer package. Other energy consumers in the process are the compressors and pumps. Equation (18) presents the relationship for calculating the thermal energy efficiency [29].

(18) $$\eta_{E}=\frac{\sum M_{products}\left(LHV_{products}\right)}{\sum Q_{h}+\sum W_{e}}$$

In the energy efficiency equation, M_*product*_ = 10235.47 kg/h is the product flow rates, $\text{LH}V_{\text{products}}=20.38\times 10^{7}\;\text{kJ}/\text{kg}$ represents low heating value of the products, Q_*h*_ = 0 kJ/h is the thermal energy required by the process, and W_*e*_ = 103 MWh is electric power consumed in the process.

#### 2.5.2 Economic evaluation

The main objective of economic evaluation is to ascertain the production cost and selling price of MeOH derived from the process. To calculate the production cost, it is necessary to evaluate two key parameters: the Total Capital Investment (TCI) and the annual operating cost (OPC) of the process. The TCI of the process is segmented into two distinct components.

1) TCI associated with the MeOH production and separation unit, and
2) TCI of the alkaline electrolyzer system.

The Lang correlation is applied for the estimation of the TCI in the MeOH production and separation unit [26].

(19) $$TCI_{Conv}=1.05f_{Lang}\sum C_{Equipment,b}\frac{I}{I_{b}}$$

The *f*_*Lange*_ of chemical processes equals to 5.93. Refer to Table B.2 for a comprehensive breakdown of this parameter. On the other hand, the following Eq. (20) is used to calculate the TCI of the electrolyzer system [30].

(20) $$TCI_{Elec}=\left(1+f_{p,Elec}\right)C_{Elec}$$

The details of the $f_{p,Elec}$ coefficient is equal to 0.5. Refer to Table B.3 for a comprehensive breakdown of this parameter. The purchase cost of the electrolyzer is determined utilizing the following scaling relationship [31].

(21) $$C_{Elec}=C_{Elec,b}{\left(\frac{P}{P_{b}}\right)}^{r}$$

The OPC of the process include the cost of renewable electricity, the cost of purchasing catalyst and process feed, labor and maintenance, etc. Table 6 provides a comprehensive summary of the OPC for the MeOH production unit.

Finally, the production cost is calculated by specifying OPC and TCI. In the subsequent stage, the Net Present Value (NPV) is employed to compute the selling price, Eq. (22). NPV break-even analysis was utilized to calculate the minimum MeOH selling price; price at which revenues received equals the costs [32].

(22) $$NPV=\sum_{tt=0}^{Life\_time}\frac{\left(S-C_{Man}\right).\left(1-t_{i}\right)-C_{dep}}{{\left(1+z\right)}^{tt}}$$

**Table 6:** Parameters of process OPC.

Parameter	Value	Unit	Ref.
*Maintenance*	*2.5*	*% of TCI*	[33]
*Insurance*	*1*	*% of TCI*	[33]
*Electrolyzer stack replacement*	*20*	*% of TCI*_*Elec*_	[30]
*Labor*	*65000*	*$/y-p*	[33]
*Operators*	*4–8*	*Persons*	[33]
*Electricity*	*49*	*$/MWh*	[34]
*CO*_*2*_	*20–60*	*$/ton*	[20,21]
*Catalyst*	*40*	*$/kg*	[33]
*Process water*	*6.5*	*$/m*^*3*^	[33]

**Table 7:** Parameters of economic evaluation.

Parameter	Value	Ref
t*t, year*	*20*	[32]
*ti*	*0.350*	[32]
*z*	*0.150*	[32]
*I*	*800.90*	[12]
*I*_*b*_	*532.90*	[12]
*C*_*Elec,b*_*, US$/MW*	*900000*	[31]
*r*	*0.65*	[31]

In Table 7, the values of the parameters used for the economic evaluation have been gathered.

In recent years, due to the turning to renewable and clean energy sources and the advancement of electrolyzer production technology, the price of electrolyzers has been decreasing. According to predictions, the purchase cost of an alkaline electrolyzer will decrease significantly by 2030 [36]. Additionally, the price of renewable resources such as solar power, hydropower, and wind power energy has been decreasing in the past years [34]. Therefore, based on the electrolyzer and renewable resources price projection, the PtM process economic analysis was done up to 2030. This analysis helps us to evaluate the viability of replacing the conventional MeOH units with PtM processes in the near future.

#### 2.5.3 PtM environmental advantages

This study uses equation (23), which indicates the amount of carbon dioxide consumed per ton of produced MeOH, to evaluate the proposed MeOH production unit's environmental features. The Equation shows the potential of the PtM process to reduce carbon dioxide emissions as well as how much carbon dioxide captured from air can be consumed in the MeOH synthesis [37].

(23) $$M_{CO_{2},Cons}=\frac{M_{CO_{2},In}-M_{CO_{2},Out}}{M_{MeOH,Out}}$$

$M_{CO_{2},In}$, $M_{CO_{2},Out}$, $M_{MeOH,Out}$ in Eq. (23) represent the amount of CO_2_ entering the process boundaries, the amount of CO_2_ exiting the process boundaries and the amount of MeOH produced in the process, respectively.

Eq. (24) was used to obtain the total amount of CO_2_ that has been avoided from being released into the atmosphere. This amount is equal to the sum of CO_2_ consumed in the PtX process, and the amount of CO_2_ produced in conventional processes that its production has been avoided by PtX processes [37].

(24) $$M_{CO_{2},Avoid}=M_{CO_{2},Conv}+M_{CO_{2},PtX}$$

(25) $$M_{CO_{2},Conv}=MeOH\;production\;capacity\;*\;{\text{CO}}_{2}\;emission/ton_{MeOH}$$

## 3 Results and discussion

### 3.1 Model validation

#### 3.1.1 Electrolyzer system

To verify the Aspen Hysys electrolyzer model and assess its accuracy, the experiment designed by Ren et al. [35] was simulated using the software. In this experiment, water and 30 % concentration KOH are introduced into the electrolyzer at a temperature of 70 °C, pressure of 16 bar, and volumetric flow rate of 2.7 m^3^/h. The anode and cathode areas of the electrolyzer are 1.4 m^2^ each, the electrolyzer consists of 45 cells and power consumption of 250 kW. A comparison of experimental and simulation results is presented in Table 8.

**Table 8:** The comparison of the electrolyzer simulation results with experimental.

***Parameter***	***Experiment*** [35]	***Hysys***	a***Relative Error, %***
***T***_***Out***_, ***°C***	*88.7*	*89.9*	*1.3*
***P***_***Out***_***, kPa***	*1600.0*	*1600.0*	*0*
***Cell voltage***, ***V***	*1.8*	*1.9*	*5.5*
***H***_***2***_***Production***, ***Nm***^***3***^***/h***	*50.0*	*54.2*	*8.4*

a Relative error = $\frac{abs\left(experiment-Simulation\right)}{experiment}\times 100$.

Considering the proximity between the experiment and simulation results, we can confidently affirm the accuracy of the electrolyzer simulation in Hysys.

#### 3.1.2 Kinetic model

Utilizing a precise and reliable kinetic model is very important in chemical reactor design. The reaction kinetics is a cornerstone in facilitating the development of an optimal reactor design. It allows for the accurate prediction of the reactor design characteristics. As mentioned, the VBF kinetic model has been used to design the MeOH synthesis reactor. The simulation results of an industrial MeOH reactor were compared to the actual industrial data to verify the MeOH synthesis reactor model. Table 9 presents the component molar flow rates in the reactor outlet stream based on the simulation results and the plant data. Also, the relative error percentages are illustrated there.

**Table 9:** The comparison of the simulation results with plant data for the component flow rates in the reactor outlet stream.

Component	Molar flow [kmol/h]		Relative error %
	Simulation	Plant data [25]	
*CO*	*181.51*	*146.14*	*19.48*
*CO*_*2*_	*516.73*	*496.85*	*3.84*
*H*_*2*_	*3136.54*	*3004.46*	*4.21*
*H*_*2*_*O*	*131.91*	*151.91*	*15.16*
*CH*_*3*_*OH*	*301.80*	*357.34*	*18.40*

**Fig. 3:**
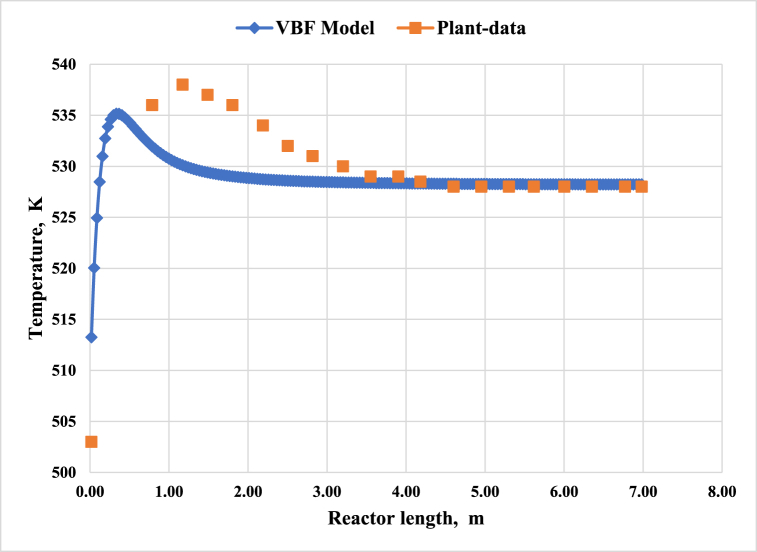
The temperature profiles along the MeOH reactor.

Fig. 3 demonstrates the reactor temperature profiles obtained from the reactor simulation and the plant data. The VBF kinetic model accurately predicts the temperature profile. The reported temperature profile along the reactor in Fig. 3 was originally provided by the reactor designer, and these values were not real operating data [25]. It is necessary to mention that the trends of the simulated temperature profile and the temperature profile reported in the reactor basic engineering document are very similar, and the maximum temperature difference is about 8 °C. Here, for validation, only the trend of the temperature variation and the reactor outlet temperature are of great importance because the stream composition at the reactor exits stream reaches its equilibrium point. Comparing the simulation results and the actual data for a MeOH synthesis reactor indicates that the proposed model has acceptable accuracy and reliability.

### 3.2 PtM Process simulation

#### 3.2.1 Hydrogen production unit

**Fig. 4:**
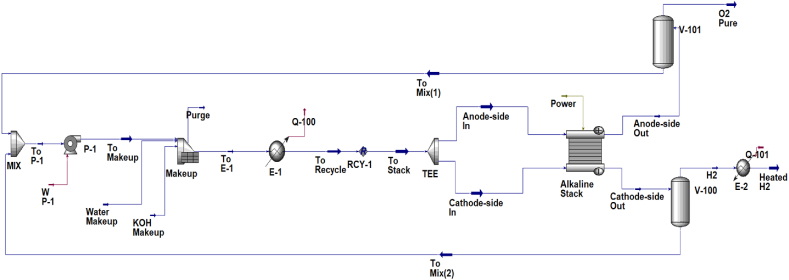
Hydrogen production unit process flow diagram.

To simulate the electrolyzer system, the Electrolyzer block of Hysys software is utilized, and the ENRTL-RK thermodynamic model is employed to predict the properties of components within the hydrogen production unit. Fig. 4 demonstrates the process flow diagram (PFD) of a hydrogen production unit. Based on the PFD, the stream of potassium hydroxide solution, at a pressure of 3000 kPa and a temperature of 55 °C, is split into two separate flows that are directed towards the cathode and anode sides. On the cathode and anode surfaces, the electrolysis reactions take place, and a mixture of hydrogen and KOH solution exits from the cathode side, while a mixture of oxygen and KOH solution exits from the anode side. KOH solution streams are first separated from the gas streams and recycled to the pump’s suction. This stream is mixed with the water and KOH make-up and enters the heat exchanger so that the temperature of the stream decreases and reaches the inlet temperature of the electrolyzer.

MeOH production has an annual capacity of 82,000 tons, necessitating the production of 15,320 tons of hydrogen annually. Using the mass balance, we calculated the water consumption required to achieve this hydrogen capacity. We considered the electrolyzer's power to be 100 MW and used the trial-and-error method to identify the optimal number of stacks for the electrolyzer's operational state. For simulation purposes, we used Aspen Hysys' built-in alkaline water electrolyzer model. Table 10 contains the details of the electrolyzer.

**Table 10:** Design parameters of the alkaline electrolyzer.

Design Parameter	Value/Comment
*Type*	*KOH Alkaline*
*Number of stacks*	*30*
*Cells per stack*	*230*^a^
*Anode active area, m*^*2*^	*4.00*^a^
*Cathode active area, m*^*2*^	*4.00*^a^
*Faraday efficiency, %*	*99.50*
*Efficiency, %*	*76.6*
*Power consumption, MW*	*100.00*
*Water consumption, kmol/h*	*964.86*
*Hydrogen production, kmol/h*	*960.29*
*T*_*out*_*, °C*	*75.2*

a Aspen-Hysys simulator default value.

#### 3.2.2 MeOH production and separation unit

**Fig. 5:**
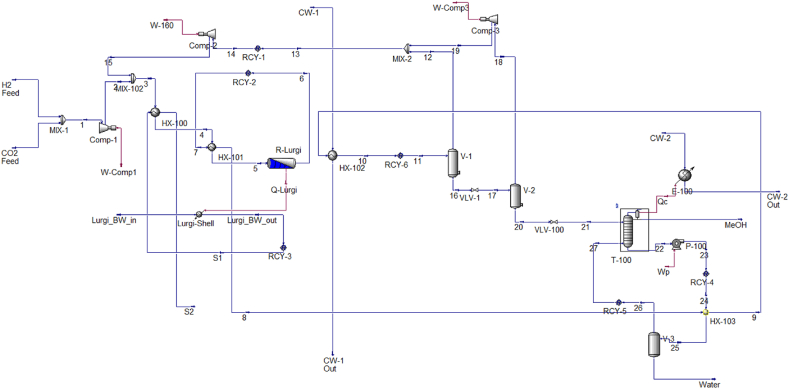
PtM process’s simulation flow-sheet in Aspen HYSYS.

Fig. 5 illustrates the simulation flow diagram of the suggested MeOH production unit.

Table 11 presents the PtM plant’s feed and product conditions at the optimum design situation. The following sections illustrate the results of the reactor optimization and heat integration.

**Table 11:** The PtM plant’s feed and product conditions at optimum design situation.

Material	Mass flow [kg/h]	
	Input to process boundaries	Output of process boundaries
*H*_*2*_	*1915.0*	*0.0*
*CO*_*2*_	*14130.3*	*49.7*
*MeOH*	*0.0*	*10235.6*
*Process water*	*0.0*	*5478.4*
*cooling water*	*10.2×10*^*5*^*@ 30 °C*	*10.2×10*^*5*^*@ 43 °C*

The table shows that the process consumes 14 tons of carbon dioxide per hour and achieves a conversion rate of 99.6 %. This data indicates that the PtM process is environmentally friendly, clean, and non-polluting. The amount of electrical energy required by the process is 102.7 MW, of which 100 MW is consumed in the electrolyzer package and 2.7 MW is related to the compressor power consumption.

##### 3.2.2.1 MeOH synthesis reactor design and optimization

This section presents the simulation and optimization results of the MeOH synthesis reactor. The kinetic model was validated in the previous sections and it was demonstrated that it accurately predicts the reaction conditions for methanol production.

##### 3.2.2.2 Optimal reactor structure and condition

After validating the developed reactor model, we utilized it to design a MeOH production reactor for PtM process. The results of the optimal design for the reactor are given in Table 12.

**Table 12:** The optimum reactor operating conditions and the design parameters.

Parameter	Value
*Tube counts*	*2883*
*T*_*in*_*, °C*	*229.9*
*P*_*in*_*, kPa*	*8469.0*
*T*_*bw*_*, °C*	*255.1*
*Pressure drops, kPa*	*197.0*
*H*_*2*_*/CO*_*2*_	*2.95*

### 3.3 Process heat integration and energy efficiency

**Fig. 6:**
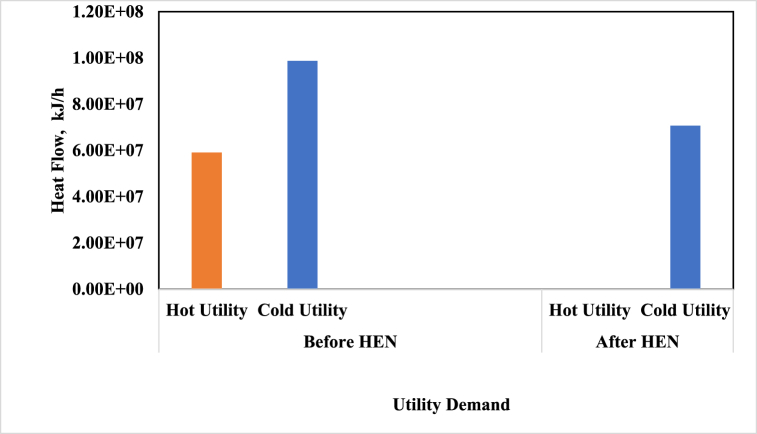
Process utility demands before and after HEN design.

The heat exchanger network (HEN) was designed after performing the pinch analysis and determining the heat transfer potential of the process streams. With the help of HEN design, the process's energy consumption has decreased significantly, causing the amount of hot utility needed by the process to reach zero, compared to 16.4 MW before applying the pinch method. Also, the implementation of the HEN design resulted in a significant reduction in cold utility consumption, from 27.4 MW to 19.6 MW as shown in Fig. 6. This change indicates a successful optimization of the system, leading to greater efficiency and cost savings. The HEN design led to a noteworthy improvement in the process's energy efficiency. Specifically, the efficiency increased from 48 % to 56 %, indicating a positive outcome.

### 3.4 Process economic evaluation

**Fig. 7:**
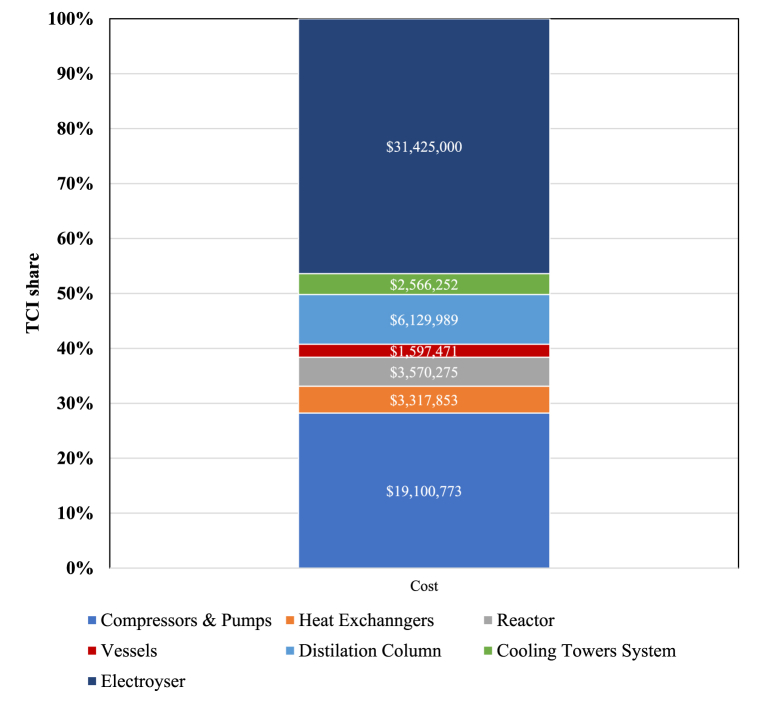
TCI breakdown of the PtM process.

In Fig. 7, the process's TCI is determined by breaking down the costs associated with the equipment.

As shown in Fig. 7, the electrolyzer TCI accounts for the largest share of the process's total cost, which increases MeOH production costs compared to conventional processes. According to Del Pozo et al., the TCI of the conventional MeOH production process, which has a capacity of 80,000 tons per year, is approximately US$ 53.03 million [31]. This figure is significantly lower than the PtM process's TCI.

**Fig. 8:**
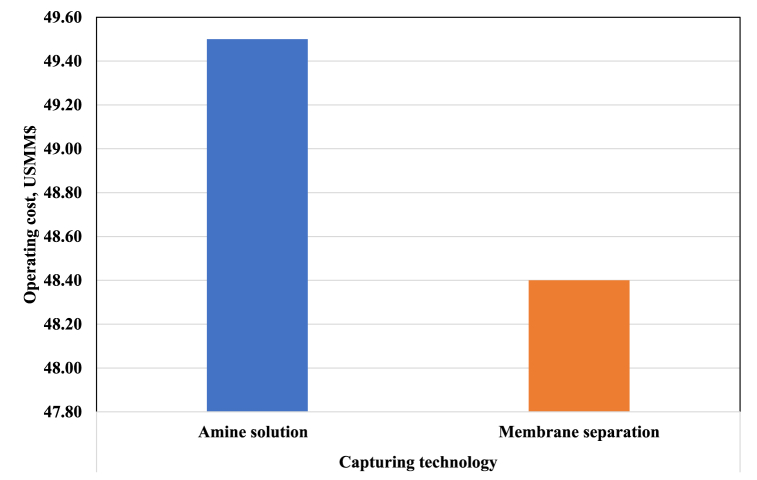
Operating costs of different CO_2_ capturing technologies.

The operational costs of the process are presented in Fig. 8. Most of these costs are related to renewable resources. As discussed in the preceding section, there exist multiple technologies that can be employed to capture CO_2_. The cost of CO_2_ capture varies depending on the type of separation technology. Here, separation with membrane and amine absorbent technologies have been compared. The price of CO_2_ obtained from the amine solution technology is higher than that capturing by membrane technology. This higher price increases the operational cost.

Based on the operating costs and total capital investment of the PtM process, the cost of producing one ton of MeOH in 2023 is estimated at US$ 643 using amine solution technology for CO_2_ capture. On the other hand, if a membrane is used for CO_2_ capture, the cost of producing one ton of MeOH is expected to be US$ 631. The NPV is set to zero to get the minimum selling price of MeOH. After deducting taxes for different CO_2_ capturing technologies, the minimum selling price of MeOH is calculated as US$ 1000 and 990 per ton, respectively. These prices are almost twice the price of MeOH in the North American market. Based on the economic indices, the PtM process still needs to be cost-competitive compared to conventional MeOH production processes. This outcome cannot encourage investors to venture into the PtM processes. We will move forward with the economic analysis of the PtM process by forecasting the total capital investment and operational costs as well as the minimum selling price of MeOH for the upcoming years to gain valuable insights.

**Fig. 9:**
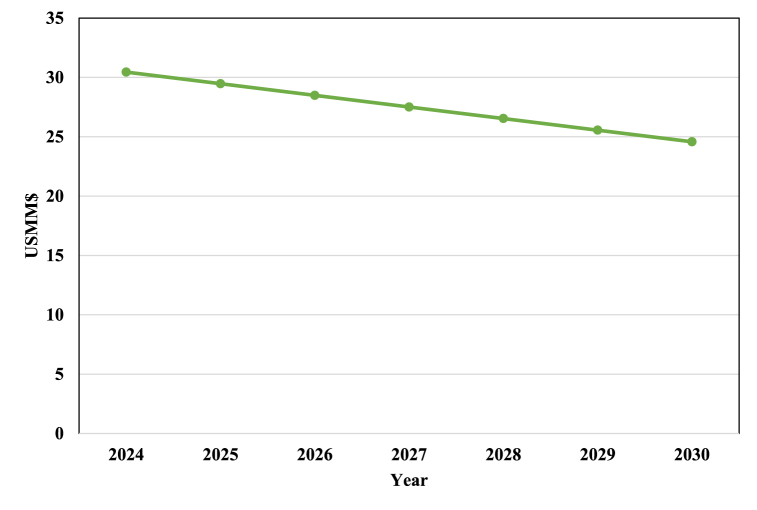
The descending trend of the alkaline electrolyzer TCI from 2024 to 2030.

Reksten et al. examined the purchase of alkaline electrolyzers in recent years and predicted the TCI of the electrolyzer for upcoming years [36]. The results project a decrease in the TCI of the electrolyzer to US$ 24.6 million by 2030, a 21.7 % decrease from 2023. According to Reksten's findings, Fig. 9 shows the trend of changes in the alkaline electrolyzer's TCI.

**Fig. 10:**
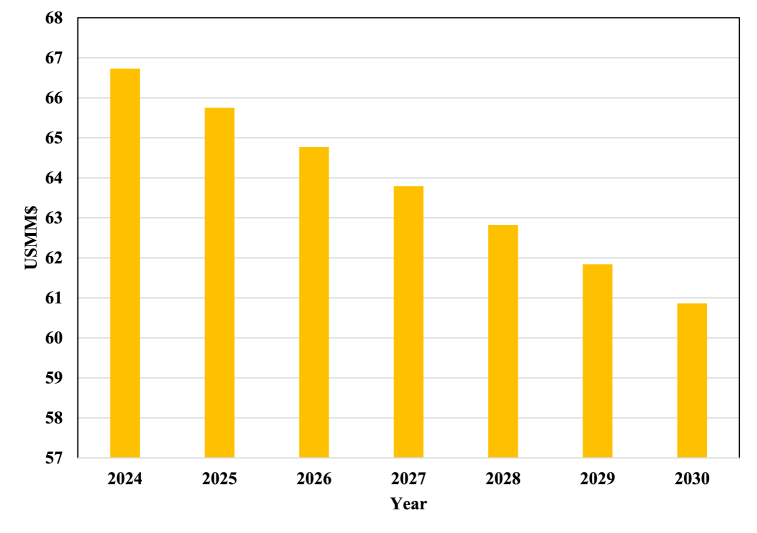
Estimation of the PtM process TCI from 2024 to 2030.

This reduction in the cost of the electrolyzer leads to a decrease in the process TCI. Fig. 10 illustrates a decreasing trend in the PtM process TCI. In Fig. 10, it is evident that the declining price of the electrolyzer in the coming years leads to a decrease in the process's TCI. We project it to reach US$ 66.7 million by 2024, a 1.4 % decrease from 2023. If this downward trend persists, we project TCI to drop to 60.8 million dollars in 2030, a 10.2 % decrease from 2023.

Despite the reduced equipment prices over the coming years, the TCI values required for the PtM process remain higher than those of conventional MeOH processes.

**Fig. 11:**
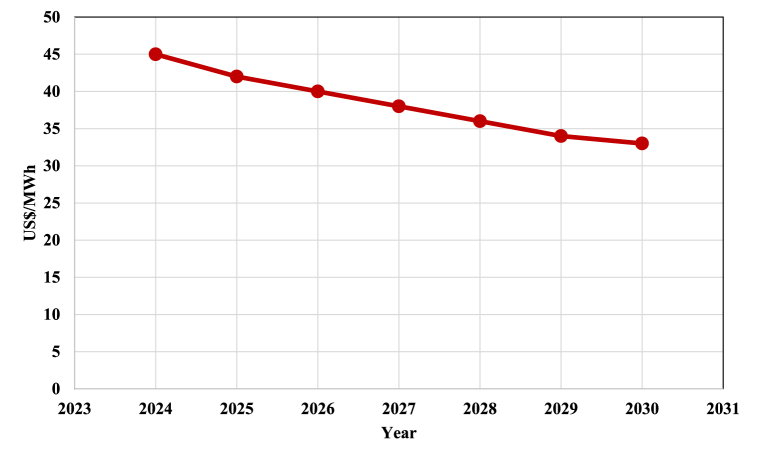
Renewable electricity price over the years 2024–2030.

Using the price data for renewable resources from 2010 to 2022 published by the International Renewable Energy Agency (IREA) [33], we have predicted the trend in the change of renewable energy prices up until 2030. It is expected that the price of renewable resources will reach US$ 33 per MWh. Fig. 11 illustrates how renewable resources' price is changing from 2024 to 2030.

**Fig. 12:**
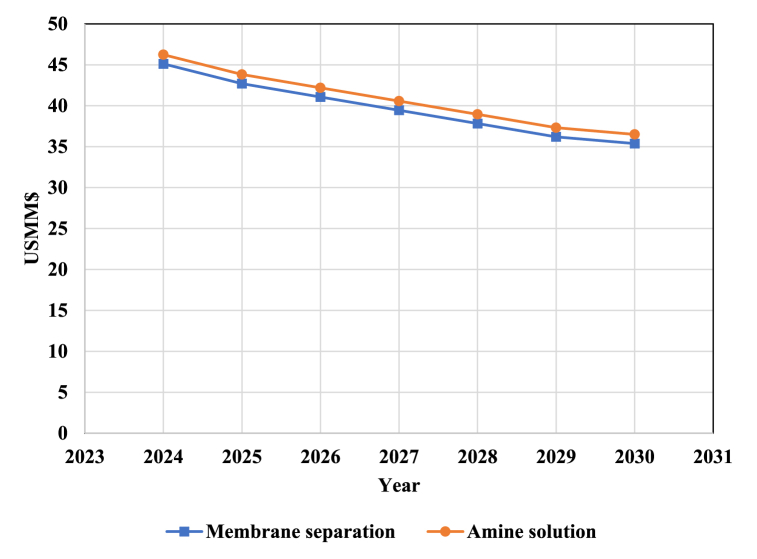
The changes in PtM process operating costs from 2024 to 2030 for two CO_2_ capturing technologies.

Since a very large part of the operating costs of the process are related to the cost of renewable energy consumed in the electrolyzer to produce hydrogen, it is obvious that the reduction of the price of renewable energy has a favorable effect on reducing the operating cost of the process. The effect of reducing the price of renewable resources on the operating costs of the process for different CO_2_ capturing technologies is shown in Fig. 12. The operating cost of the process for amine separation technology and membrane separation in 2030 is estimated at 36.5 and 35.4 million dollars, respectively.

**Fig. 13:**
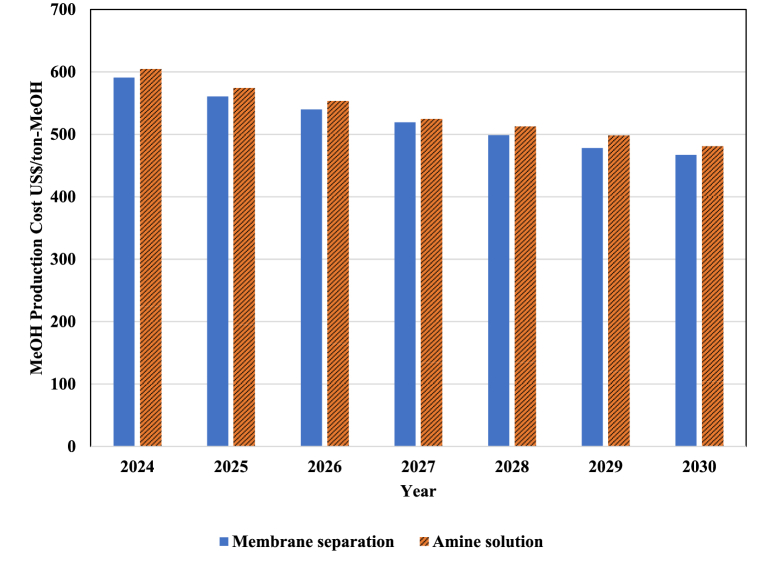
The variation of MeOH production Costs from 2024 to 2030.

Considering that the cost of MeOH production is calculated using TCI and OPC, then the reduction in TCI and OPC process leads to the reduction of MeOH production cost. The changes in MeOH production cost for different CO_2_ capturing technologies are shown in Fig. 13. Considering the decreasing trend of TCI and OPC processes, it is expected that the cost of MeOH production for amine absorption technology will decrease from US$ 643 in 2023 with a 25.3 % decrease to US$ 480 in 2030. This is while with membrane absorption technology, the cost of MeOH production in 2030 will decrease by 26 % to US$ 631 compared to 2023.

**Fig. 14:**
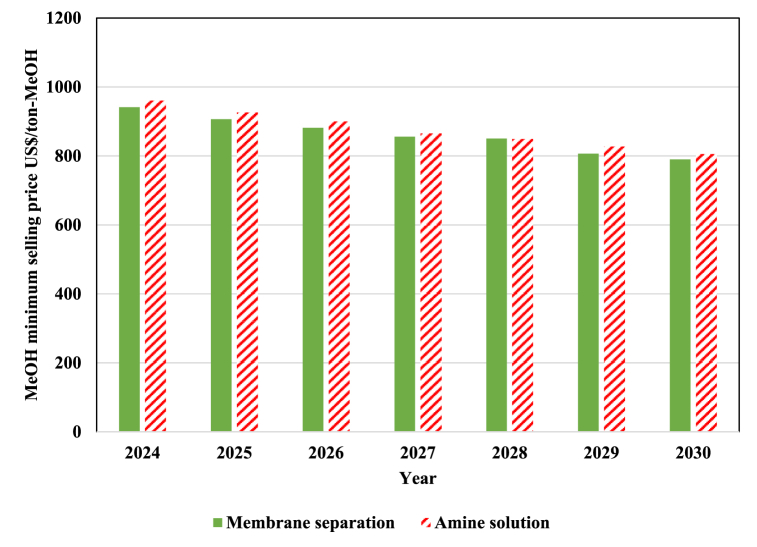
The projection of MeOH minimum selling price in the years 2024–2030.

The decrease in the cost of MeOH production leads to a decrease in the minimum selling price of MeOH, which is shown in Fig. 14 for different CO_2_ absorption technologies. The minimum selling price in the process of converting to MeOH with CO_2_ capturing technology with amine solution will reach US$ 805 in 2030, which is 1.6 times more than the global price of MeOH. On the other hand, with membrane separation, the minimum selling price is expected to reach US$ 790 by 2030, which is far from the world price of MeOH.

The data presented in Fig. 14 suggests that despite the downward trend in the electrolyzer's total cost of ownership and the process's operating cost, the minimum selling price of MeOH obtained from the PtX process appears to remain above the current price of MeOH produced via conventional processes.

### 3.5 Environmental evaluation

**Fig. 15:**
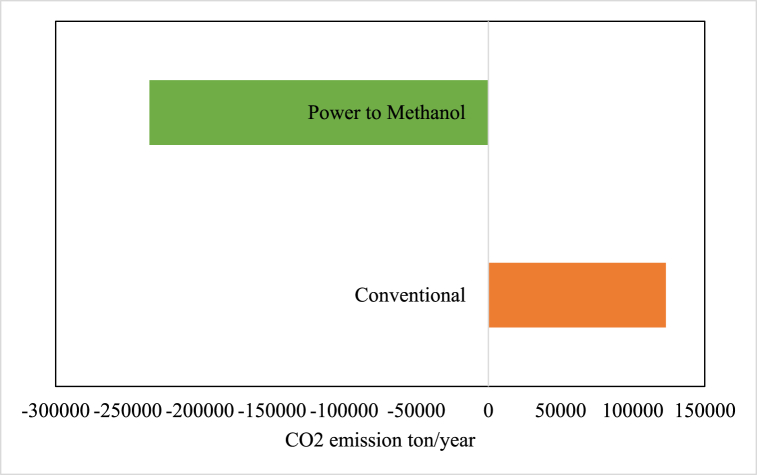
CO_2_ emissions of the proposed PtM process and the conventional MeOH production process.

The advantage of PtX processes over conventional methods for producing chemical products is their environment-friendly nature and sustainable practices. Although PtX processes are considered an expensive technology, they can prove to be very effective in the future from an environmental perspective, and help eliminate greenhouse gases in the atmosphere. As mentioned in the economic evaluation section, these processes must still compete economically. This study shows that the developed PtM process consumes 112,000 tons of CO_2_ per year, equal to 1.4 tons of CO_2_ per ton of MeOH production. On a global scale, the production capacity of MeOH is 100 million tons. If we assume that all the MeOH produced in the world is produced from PtM processes, it can lead to the consumption of 140 million tons of CO_2_, which is a considerable amount. While the conventional processes of MeOH production were releasing 150 million tons of carbon dioxide, this new technology can prevent the release of this vast amount of pollution into the atmosphere. According to the analysis, the developed process can avoid the annual release of 235,000 tons of CO_2_. Fig. 15 shows the proposed PtM process's CO_2_ emissions in comparison with the conventional MeOH production process with the same capacity.

## 4 Conclusion

In this study, we conducted research on the development and evaluation of a PtM process. We analyzed it from technical, economic, and environmental perspectives and used Aspen HYSYS software to perform the process simulation. Our assessments revealed that in the optimal state, the process exhibited an energy efficiency of 56 %. Additionally, the developed PtM process consumes 1.4 kg-CO_2_/kg-MeOH. Based on the economic analysis, we estimated that the costs of producing MeOH in the pivotal year of 2023, utilizing membrane technology and amine solution for CO_2_ capturing, were expected to be US$ 631 and 643, respectively. We also estimated the cost of production and the minimum selling price of MeOH obtained from this process using the forecasts made on the electrolyzer's TCI and the price of renewable resources until 2030. Despite the downward trend in the electrolyzer's total cost of ownership and the process's operating cost, the minimum selling price of MeOH obtained from the PtX process appears to remain above the current price of MeOH produced via conventional processes. This study highlights the potential of the developed PtM process in reducing CO_2_ emissions. The process consumes 112,000 tons of CO_2_ per year, equal to 1.4 tons of CO_2_ per ton of MeOH production. Assuming that all the MeOH produced in the world is produced from PtM processes, it can lead to 140 million tons of CO_2_ consumption, significantly reducing global emissions. This new technology can prevent the release of 150 million tons of carbon dioxide into the atmosphere, a vast amount of pollution. According to the analysis, the developed process can avoid the annual release of 235,000 tons of CO_2_, contributing significantly to environmental sustainability.

According to the results of the economic evaluation section of the process, the economic bottleneck of the PtM process is the cost of the electrolyzer, the price of electricity from renewable sources, and the cost of carbon dioxide capture. It is clear that the decrease in the costs of electrolyzers and renewable energy sources, coupled with the availability of more affordable CO_2_-capturing technologies, can contribute to the enhanced competitiveness of PtX processes. However, it is crucial to consider that the forecasts indicate a limited reduction in the prices of electrolyzers and renewable energy sources. If progress is made in improving the current trend, achieving zero greenhouse gas emissions by 2050 will be possible. However, to ensure this happens, it is necessary for some strict legal measures to be implemented, such as reducing or eliminating taxes on PtX processes and increasing fines and taxes for high-carbon-emitting units.

The research indicates that PtX technology-based processes have enormous potential for sustainability. Although hydrogen production using an electrolyzer is attractive and promising from an environmental perspective, the high rate of degradation of the electrodes and catalysts in this method poses a technical barrier to industrialization. Apart from lowering the cost, it is crucial to enhance the stability of the electrolyzer, paving the way for the PtX processes to emerge as a viable alternative for chemical compound production in the future.

The processes are still in the conceptual stage and have not been implemented yet, they may provide a suitable solution for producing chemical products once fossil resources are depleted. However, to realize their full potential, these processes need to become more competitive and receive greater attention. The promising results suggest that PtX processes have the potential to become large-scale industrial processes in the near future, which would be a significant achievement for sustainable development.

## CRediT authorship contribution statement

**Ali Pakdel:** Writing – original draft, Visualization, Software, Methodology. **Reza Eslamloueyan:** Writing – review & editing, Supervision, Project administration.

## Funding and sponsors

The authors declare no funding from any organization or sponsor in this article.

## Declaration of competing interest

The authors declare that they have no known competing financial interests or personal relationships that could have appeared to influence the work reported in this paper.

## Figures and Tables

**Table A.1:** Properties for streams of the electrolyzer system.

Stream	Properties			Components mole fraction			
	T, °C	P, kPa	Flowrate, kmol/h	H_2_	O_2_	H_2_O	KOH
*To P-1*	*76.1*	*3004.9*	*56634.2*	*0.0000*	*0.0001*	*0.9016*	*0.0983*
*To Makeup*	*76.1*	*3027.0*	*56634.2*	*0.0000*	*0.0001*	*0.9016*	*0.0983*
*Water Makeup*	*76.1*	*3027.0*	*973.1*	*0.0000*	*0.0000*	*1.0000*	*0.0000*
*KOH Makeup*	*76.1*	*3027.0*	*0.0*	*0.0000*	*0.0000*	*0.0000*	*1.0000*
*Purge*	*76.1*	*3027.0*	*0.0*	*0.0000*	*0.0000*	*0.0000*	*0.0000*
*To E-1*	*76.1*	*3027.0*	*57607.2*	*0.0000*	*0.0001*	*0.9032*	*0.0967*
*To Recycle*	*55.0*	*3027.0*	*57607.2*	*0.0000*	*0.0001*	*0.9032*	*0.0967*
*Anode-side In*	*55.0*	*3027.0*	*20162.5*	*0.0000*	*0.0001*	*0.9032*	*0.0967*
*Cathode-side In*	*55.0*	*3027.0*	*37444.7*	*0.0000*	*0.0001*	*0.9032*	*0.0967*
*Anode-side Out*	*75.2*	*3004.9*	*27398.9*	*0.0000*	*0.0176*	*0.9112*	*0.0712*
*Cathode-side Out*	*75.2*	*3004.9*	*30688.5*	*0.0313*	*0.0000*	*0.8507*	*0.1180*
*O*_*2*_	*75.2*	*3004.9*	*486.2*	*0.0010*	*0.9866*	*0.0124*	*0.0000*
*To Mix (1)*	*75.2*	*3004.9*	*26912.6*	*0.0000*	*0.0001*	*0.9274*	*0.0724*
*H*_*2*_	*75.2*	*3004.9*	*950.9*	*0.9995*	*0.0000*	*0.0005*	*0.0000*
*To Mix (2)*	*75.2*	*3004.9*	*29737.6*	*0.0003*	*0.0000*	*0.8779*	*0.1217*
*Heated H*_*2*_	*80.0*	*3000.0*	*950.9*	*0.9995*	*0.0000*	*0.0005*	*0.0000*

**Table A.2:** Properties for streams of the methanol production unit.

Stream	Properties			Components mole fraction				
	T, °C	P, kPa	Flowrate, kmol/h	H_2_	CO_2_	MeOH	H_2_O	CO
*H*_*2*_*Feed*	*80.0*	*3000.0*	*950.0*	*1.0000*	*0.0000*	*0.0000*	*0.0000*	*0.0000*
*CO*_*2*_*Feed*	*80.0*	*3000.0*	*321.1*	*0.0000*	*1.0000*	*0.0000*	*0.0000*	*0.0000*
*1*	*75.2*	*3000.0*	*1271.1*	*0.7474*	*0.2526*	*0.0000*	*0.0000*	*0.0000*
*2*	*219.8*	*8472.0*	*1271.1*	*0.7474*	*0.2526*	*0.0000*	*0.0000*	*0.0000*
*3*	*99.9*	*8472.0*	*7798.2*	*0.8848*	*0.1085*	*0.0046*	*0.0010*	*0.0011*
*4*	*179.4*	*8470.8*	*7798.2*	*0.8848*	*0.1085*	*0.0046*	*0.0010*	*0.0011*
*5*	*229.9*	*8468.7*	*7798.2*	*0.8848*	*0.1085*	*0.0046*	*0.0010*	*0.0011*
*6*	*255.3*	*8271.6*	*7159.1*	*0.8299*	*0.0736*	*0.0496*	*0.0458*	*0.0012*
*8*	*203.0*	*8270.9*	*7159.1*	*0.8299*	*0.0736*	*0.0496*	*0.0458*	*0.0012*
*9*	*125.9*	*8269.4*	*7159.1*	*0.8299*	*0.0736*	*0.0496*	*0.0458*	*0.0012*
*10*	*54.6*	*5265.6*	*7159.2*	*0. 8299*	*0.0736*	*0.0496*	*0.0458*	*0.0012*
*12*	*54.1*	*7265.6*	*6509.8*	*0.9124*	*0.0799*	*0.0052*	*0.0012*	*0.0013*
*13*	*55.1*	*7265.6*	*6518.9*	*0.9114*	*0.0806*	*0.0055*	*0.0012*	*0.0013*
*15*	*74.6*	*8472.0*	*6527.2*	*0.9116*	*0.0805*	*0.0055*	*0.0012*	*0.0013*
*16*	*54.1*	*7265.6*	*649.3*	*0.0025*	*0.0101*	*0.4947*	*0.4927*	*0.0000*
*17*	*55.9*	*1000.0*	*649.3*	*0.0025*	*0.0101*	*0.4947*	*0.4927*	*0.0000*
*18*	*54.5*	*150.0*	*9.1*	*0.1760*	*0.5973*	*0.1845*	*0.0419*	*0.0003*
*20*	*54.5*	*150.0*	*640.3*	*0.0000*	*0.0018*	*0.4991*	*0.4991*	*0.0000*
*21*	*54.4*	*115.0*	*640.3*	*0.0000*	*0.0018*	*0.4991*	*0.4991*	*0.0000*
*22*	*107.0*	*130.0*	*991.5*	*0.0000*	*0.0000*	*0.0022*	*0.9978*	*0.0000*
*23*	*107.0*	*136.0*	*1001.5*	*0.0000*	*0.0000*	*0.0022*	*0.9978*	*0.0000*
*25*	*107.6*	*132.7*	*1001.5*	*0.0000*	*0.0000*	*0.0022*	*0.9978*	*0.0000*
*26*	*107.0*	*130.0*	*697.7*	*0.0000*	*0.000*	*0.0031*	*0.9969*	*0.0000*
*MeOH*	*43.9*	*105.0*	*329.0*	*0.0000*	*0.0003*	*0.9806*	*0.0191*	*0.0000*
*Water*	*107.0*	*130.0*	*313.6*	*0.0000*	*0.0000*	*0.0001*	*0.999*	*0.0000*
*CW-1*	*30.0*	*1015.0*	*29000.0*	*0.0000*	*0.0000*	*0.0000*	*1.0000*	*0.0000*
*CW-1 Out*	*43.9*	*1006.6*	*29000.0*	*0.0000*	*0.0000*	*0.0000*	*1.0000*	*0.0000*
*CW-2*	*30.0*	*1015.0*	*27500.0*	*0.0000*	*0.0000*	*0.0000*	*1.0000*	*0.0000*
*CW-2 Out*	*42.3*	*1013.5*	*27500.0*	*0.0000*	*0.0000*	*0.0000*	*1.0000*	*0.0000*
*Lurgi_BW_in*	*255.0*	*4249.9*	*432.4*	*0.0000*	*0.0000*	*0.0000*	*1.0000*	*0.0000*
*Lurgi_BW_out*	*255.0*	*4249.9*	*432.4*	*0.0000*	*0.0000*	*0.0000*	*1.0000*	*0.0000*
*S2*	*100.5*	*4249.4*	*432.4*	*0.0000*	*0.0000*	*0.0000*	*1.0000*	*0.0000*

**Table B.1:** Equipment cost data

Equipment	s	unit	a	b	n	ref
*Pump*	*Flow rate*	*L/s*	*8000*	*240*	*0.9*	[32]
*Explosion motor*	*Power*	*kW*	*−1100*	*2100*	*0.6*	[32]
*Compressor*	*Power*	*kW*	*580000*	*20000*	*0.6*	[32]
*Reactor*	*Volume*	*m*^*3*^	*61500*	*32500*	*0.5*	[32]
*Heat exchanger*	*Area*	*m*^*2*^	*28000*	*54*	*1.2*	[32]
*Vessels*	*Weight*	*kg*	*11600*	*34*	*0.85*	[32]
*Distillation column*	*Weight*	*kg*	*11600*	*34*	*0.85*	[32]
*Sieve tray*	*Diameter*	*m*	*130*	*440*	*1.8*	[32]
*Cooling towers*	*Flow rate*	*L/s*	*170000*	*1500*	*0.9*	[32]

**Table B.2:** Lang factor breakdown

Parameter	Value	Ref.
*equipment delivery*	*1.00*	[26]
*Installation*	*0.47*	[26]
*electrical*	*0.11*	[26]
*piping*	*0.68*	[26]
*control and instrumentation*	*0.36*	[26]
*buildings*	*0.18*	[26]
*service facilities*	*0.70*	[26]
*yard improvement*	*0.10*	[26]
*supervision and engineering*	*0.33*	[26]
*construction*	*0.41*	[26]
*Contingency*	*0.44*	[26]
*Contractor’s fee*	*0.26*	[26]
*working capital*	*0.89*	[26]
$f_{Lang}$	*5.93*	[26]

**Table B.3:** Electrolyzer TCI factor breakdown.

Parameter	Value	Ref.
*Delivery*	*0.15*	[30]
*Site and construction*	*0.19*	[30]
*engineering*	*0.01*	[30]
*Contingency*	*0.15*	[30]
$f_{p,Elec}$	*0.50*	[30]

## Appendix B

The purchase cost of process equipment is calculated from the following formula [32].

Eq. B1 $$C_{p}=a+b{\left(s\right)}^{n}$$

which s in Eq. (B1) is size factor of equipment and a, b, and c constant for each equipment gathered in Table B.1 which Chemical Engineering plant cost index = 532.9 [32].
